# Beyond pain relief: a thematic exploration of informal caregiver experiences and needs following SPING block treatment in geriatric hip fracture patients

**DOI:** 10.1007/s41999-026-01432-y

**Published:** 2026-03-05

**Authors:** Miriam W. A. van der Velden, Nick J. G. Visschers, Miriam C. Faes, Bart Spaetgens, Eline L. Brundel, Marieke H. J. van den Beuken-van Everdingen, Arnela Suman

**Affiliations:** 1 Department of Pain Medicine and Palliative Care, Amphia Hospital, Breda, The Netherlands; 2 Department of Geriatrics, Amphia Hospital, Breda, The Netherlands; 3 Geriatrics Section, Department of Internal Medicine, Maastricht University Medical Center, Maastricht, The Netherlands; 4 CAPHRI Research Institute, Maastricht University Medical Center, Maastricht, The Netherlands; 5 Center of Expertise in Palliative Care, Maastricht University Medical Center, 6229 HX Maastricht, The Netherlands

**Keywords:** Palliative care, Hip fracture, Non-operative pain management, SPING, Qualitative research

## Abstract

**Purpose:**

To explore informal caregivers’ experiences and preferences regarding decision-making and care processes following the Spinal Phenol IN Glycerol (SPING) block as non-operative pain management in frail older care recipients with a hip fracture. The aim is to improve the care process of the SPING block and improving communication with and support for informal caregivers.

**Methods:**

A single-center qualitative study was conducted at Amphia Hospital (the Netherlands) using semi-structured interviews. Informal caregivers of frail, older patients with hip fracture treated with SPING block were purposively sampled until saturation was reached. Interviews were audio-recorded and transcribed verbatim for thematically analysis.

**Results:**

Four dominant themes were identified: (1) Pain relief as a core treatment priority; (2) Complex decision-making and the caregiver’s moral burden; (3) Emotional toll and coping with witnessing decline; and (4) Shifting roles and long-term caregiving burden. SPING block was viewed as an acceptable, often preferred non-operative pain management. Informal caregivers valued rapid analgesia and the possibility of early discharge yet described insufficient communication and uncertainty around long-term effects. The caregiving burden extended beyond hospital discharge, with many reporting distress and role conflict.

**Conclusion:**

Informal caregivers prioritize effective analgesia, compassionate communication and continuity of care when navigating decisions about SPING block in the context of frailty and end-of-life care. These findings underscore the importance of integrated, multidisciplinary protocols that facilitate timely decision-making, align with patient goals and address caregiver needs across care settings. Prospective studies are warranted to evaluate patient-centered outcomes of SPING block and its long-term analgesic effects.

## Key summary points

**Aim:**

To improve the care process of the SPING block and improving communication with and support for informal caregivers.

**Findings:**

SPING block was viewed as an acceptable, often preferred non-operative pain management. Informal caregivers valued rapid analgesia and the possibility of early discharge yet described insufficient communication and uncertainty around long-term effects.

**Message:**

Informal caregivers prioritize effective analgesia, compassionate communication and continuity of care when navigating decisions about SPING block in the context of frailty and end-of-life care.

## Introduction

Hip fractures represent a growing global health concern due to the rapidly ageing population [1, 2]. Among frail, older adults with multimorbidity, the incidence of hip fractures is particularly high and is frequently associated with functional decline [3, 4]. For many patients, a hip fracture may mark the beginning of a palliative trajectory [5–9]. While surgery remains the gold standard of care for patients with a hip fracture, its risks and benefits become more complex in the context of advanced frailty [5–9]. Perioperative complications such as delirium, immobility, prolonged hospitalization and mortality are substantial. Additionally, the chances of regaining the level of pre-fracture mobility or functionality are often limited [8, 9]. As a result, surgery may not align with the treatment goals of patients in the palliative phase, which often emphasize comfort, dignity, quality of life and remaining in a familiar environment [5], while minimizing treatment burden [10]. Nonoperative management (NOM) is increasingly considered a viable alternative for surgery in frail, older adults with a hip fracture with limited prognosis and rehabilitation potential. Although NOM may be appropriate within an advanced care planning context, observational data indicate that short-term mortality is generally higher than after surgery [11]. One of the major challenges remains adequate and sustained control of pain [12]. High-dose opioid regimens are often necessary, but come with well-known side effects such as sedation, delirium, and reduced ability to communicate, which may further compromise quality of life in the palliative phase for patients [11, 12].

The Spinal Phenol IN Glycerol (SPING) block, as NOM for hip fractures, is a single-shot, intrathecal neurolytic procedure, developed to offer rapid and sustained pain relief in patients for whom surgery is not a preferred treatment option [12–14]. Preliminary observations suggest that the SPING block provides adequate pain relief, supports ease of care and can facilitate the ability to sit upright, reduce opioid requirements, support timely discharge and the possibility of spending meaningful time with loved ones in a familiar environment [13, 14]. Therefore, SPING block may offer a meaningful addition to the palliative pain management arsenal in hip fracture care. However, while SPING block treatment is increasingly applied, little is known about how it is experienced and evaluated by informal caregivers. Informal caregivers are particularly relevant given that up to 42% of frail, older patients diagnosed with hip fracture present with cognitive impairment, limiting their capacity to fully participate in complex decision-making concerning their treatment [15]. In these cases, informal caregivers play a crucial role in representing the patient’s preferences, navigating uncertainty and co-determining care and treatment goals [16]. Their experiences offer unique insights into the practical and emotional implications of care decisions in late life [17]. Understanding how informal caregivers perceive and evaluate SPING block treatment is essential to ensure that care remains aligned with patient needs,values and treatment goals. While the broader palliative care literature highlights the importance of incorporating caregiver perspectives [18], such knowledge is lacking in the context of NOM and SPING block for hip fractures in frail, older adults.

This study explores how informal caregivers experience shared decision-making (SDM) and the care process surrounding SPING block treatment for hip fractures in frail, older adults, with the aim of informing more personalized and goal-oriented NOM, specifically SPING block treatment. This information will contribute to better alignment between clinical practice and the values of these patients and their informal caregivers.

## Methods

A qualitative study was conducted using semi-structured interviews among informal caregivers of frail, older adults (>/= 70 years) with hip fracture who received SPING block as NOM. All patients had been treated at Amphia Hospital, a large teaching hospital in the Netherlands. Institutional data indicate that approximately 570 hip fractures are diagnosed annually at this centre, of which about 8.4% are managed using a non-operative approach. The decision for the SPING block had been made in accordance with Amphia’s standardized care pathway for hip fractures in older patients. Within this pathway, all patients aged ≥ 70 years with a hip fracture are routinely assessed by a geriatrician. The geriatrician performs a structured frailty evaluation across somatic, psychological, functional, and social domains. Based on this assessment and the outcome of the SDM with the patient and informal caregivers, SPING block as NOM is considered for those in whom surgical treatment is expected to provide limited benefit due to frailty, advanced age, multimorbidity, or restricted functional reserve.

The institutional review board of Amphia approved the study and determined that it was not subject to the Medical Research Involving Human Subjects Act (WMO). This article was written according to the Consolidated criteria for reporting qualitative research (COREQ) checklist [19].

### Participants and recruitment

The informal caregiver was defined as the non-professional who was most involved in caring for the patient, assisting with at least one personal or instrumental activity of daily living, monitoring the patient at least two times per week and playing an active role in the treatment decision. An active role is defined as participating in discussions or meetings about treatment decisions and/or providing consent on behalf of the patient. The method of purposive sampling was applied to the recruitment process, which involves a deliberate selection of subjects, to obtain a full view of the experiences of informal caregivers [20]. Inclusion required that the decision for SPING block had been made according to Amphia’s standardized care pathway for hip fracture in older patients. Only caregivers who had participated in the SDM process prior to inclusion were eligible.

Informal caregivers were purposively sampled based on characteristics of the patient under their care, i.e. age, gender, cognitive status, and pre-fracture living situation.

Informal caregivers were contacted for recruitment by telephone by a member of the research team. They were informed that participation was voluntary and that they could withdraw at any time without consequences. Written informed consent was obtained prior to the start of the audio-recorded interview.

### Data collection

All interviews were conducted in person, by a single researcher (EB). This approach was deliberately chosen to ensure consistency between interviews and minimize interviewer variation. We used a semi-structured topic guide, co-developed by the research team (EB, MvdV and AH) and informed by literature insights from the FRAIL HIP and PENG studies [5, 10, 21]. It was developed as a tool to explore key themes and was iteratively refined throughout the data collection process. Based on insights gained from ongoing interviews, the guide was continuously adjusted. For example, the introductory framing of the shared-decision-making section was expanded to a more detailed and empathic narrative, enhancing contextual clarity for participants. In addition, broad questions about the decision not to operative was subdivided into multiple probes, allowing for greater depth and consistency.

Interviews were conducted at a location of the participant’s preference. Non-verbal cues were captured through field notes. All interviews were audio-recorded and transcribed verbatim. Transcripts were pseudonymized by assigning random participant numbers and anonymizing identifiable information. Data collection continued until thematic saturation was reached, defined as the point at which no new conceptual themes emerged from subsequent interviews. A few clinical outcomes of the patients, including survival status and time to death following SPING block treatment, were also recorded.

### Data analysis

Thematic analysis was conducted on the transcripts in accordance with the Braun & Clarke's model using the software program Atlas.ti 24.2.1 [22, 23]. Seven initial interviews were independently coded by two researchers (NV and EB), to ensure intercoder agreement. Disagreements were discussed until consensus was reached. In case of uncertainty, a third researcher (AH) reviewed the coding and contributed to the final coding decision. Quotations selected for publication were translated into English.

## Results

Of the 22 eligible caregivers, fourteen caregivers were approached for participation. Of these, two declined because the interview would be emotionally too difficult. Twelve informal caregivers (9 men and 3 women) were interviewed, between 11 and 40 months after their relative had received SPING block treatment. One interview involved both the patient's partner and daughter, which explains why there were more caregivers interviewed than patients treated. All were family members of the patient. This resulted in a response rate of 85%. On average, the interviews lasted one hour and 43 min.

Table 1 summarizes the socio-demographic and health characteristics of caregivers and their care recipients (patients). Most patients were male, with a mean age of 87 years. Cognitive impairment, most commonly dementia, was present in 73% of patients. Pre-fracture living situations included community dwelling, nursing home and geriatric rehabilitation care. Ten patients had passed away at time of the interview, on average 101 days after SPING block treatment.

**Table 1:** Socio-demographic and health characteristics of caregivers and their care recipients

Characteristics	Caregivers (*n* = 12)	Care recipients (patients) (*n* = 11)*
Mean age (range)	Unknown	87 years (75–96)
Male	9 (75%)	7 (64%)
Relationship to patient		N/A
Spouse	2	
Child	10	
Cognitive impairment	N/A	
None		3
Dementia		8
Pre fracture living situation	N/A	
Community dwelling		5
Nursing home		5
Geriatric rehabilitation care		1
Time between SPING block treatment and interview (mean months; range)	21 (11–40)	N/A

*In one case, both a spouse and adult child participated in a joint interview

*N/A* Not applicable

Thematic analysis yielded four major themes, i.e. (1) Pain relief as a core treatment priority; (2) Complex decision-making and the caregiver’s moral burden; (3) Emotional toll and coping with witnessing decline; and (4) Shifting roles and long-term caregiving burden.

## Pain relief as a core treatment priority

### Alleviation of pain

Pain alleviation emerged as the most frequently reported and prioritized treatment goal among informal caregivers. They emphasized that pain relief was not merely a clinical objective, but a personal priority, as it directly affected the patient’s quality of life and daily comfort. Informal caregivers regarded effective pain management as essential for reducing suffering and preserving dignity. SPING block was viewed as an acceptable and preferable treatment option. The decision to opt for SPING block was thus motivated not only by medical considerations.

For some caregivers, the patient's pain became all-encompassing, impacting the patient’s fundamental daily activities such as eating, sleeping, and the ability to provide basic care and comfort. Patients experienced severe pain during hospital admission prior to SPING block treatment. Particularly during movement, which was reported as highly distressing. Experiences with the SPING block varied widely among caregivers. For some patients, it brought significant relief and restored comfort; for others, its effects were more modest. In these cases, patients continued to experience residual pain despite treatment with SPING block, which limited improvement of their quality of life. The persistent pain often required additional analgesia.

*“.. Everything was about that… I think he couldn’t eat because of the pain, I think he couldn’t sleep because of the pain, I think he didn’t want to sit in the chain. Couldn’t sit because of the pain… So actually, it was all-encompassing… He just lay there, and nothing else was possible anymore, so for him, I think, life just stopped… He even begged for it, like, ‘let me go.’”*

*“It was really miraculous, I thought. She had the phenol injection and after that she was sitting upright in bed, without pain. [smiles] Yeah, that was really something special to witness. But yeah, her memory is obviously also something that plays a role, you notice that afterwards. I mean, in that moment you all think like, okay.. but she really had to get used to the fact that some things just weren’t possible anymore.”*

### Minimizing hospital stay and the positive effect on emotional distress

Informal caregivers emphasized the importance of minimizing the duration of hospital admission. Prolonged hospitalization was perceived as potentially exacerbating both physical and psychological discomfort. The length of hospital stays varied considerably and caregivers’ experiences with length and quality of stay appeared to be strongly influenced by the degree of coordination between healthcare providers. In some cases, involvement of multiple disciplines of healthcare providers was perceived as delaying treatment initiation; however, in other cases, the process was described as efficient, with interventions carried out on the same day, facilitating early discharge to a nursing home.

A short hospital stay was closely linked to the overarching desire to be reunited with loved ones. Family and social connections emerged as central to the treatment experience and were frequently cited as a key factor influencing SDM. Informal caregivers emphasized that the presence of loved ones provided not only emotional support but was also integral to the patient’s perceived quality of life. Reunion of the parents after hospitalization, even amid evident physical decline, was associated with emotional relief, a sense of togetherness, and affirmation of care goals.

*“Because in the end, when he arrived there. Well, that image of him crying, together with my mother.. and I think they lay in each other’s arms for five minutes, like, “oh, we’re together again.” Yeah... [smiles] How wonderful is that?”*

## Complex decision-making and the caregiver’s moral burden

SDM in the context of SPING block treatment was described as multifaceted, often occurring under time pressure and in emotionally demanding circumstances. Due to the prevalence of cognitive impairment among patients, decision-making was frequently delegated to informal caregivers, who carried significant responsibility in determining what course of action aligned best with the patient’s values and best interests. This responsibility had a clear emotional impact: informal caregivers often experienced uncertainty and doubt about whether they were making the right choice, which could be distressing. In the absence of formal advance care plans, decisions were typically based on previously expressed general preferences, such as a wish not to undergo invasive treatment or resuscitation, though these were not always easily translated into specific clinical choices.

Caregivers’ experiences with the decision-making process varied considerably. While some described open, supportive communication with healthcare professionals, others encountered conflicting perspectives or felt that their concerns were inadequately addressed. A strong preference was expressed for clear, empathetic, and timely communication particularly regarding prognosis, palliative options, and the implications of choosing SPING block as NOM. Written information was considered helpful but insufficient on its own; caregivers emphasized the need for in-depth, face-to-face dialogue to discuss uncertainties and explore treatment goals.

For some, the decision to proceed with SPING block was experienced as a ‘second-best’ option to surgery, driven more by the limitations imposed by the patient’s frailty and poor rehabilitation potential than by personal conviction. In several cases, the perceived urgency of decision-making, combined with strong recommendations from clinicians, created a sense of coercion. Although the decision-making process at times led to retrospective doubt about whether it truly reflected the family’s preferences, most caregivers ultimately regarded the choice for SPING block as appropriate and acceptable particularly when supported by sufficient time, explanation, and alignment with the patient’s condition, values, and the limited treatment alternatives available.

Importantly, decision-making extended beyond the hospital setting. General practitioners and nursing home physicians played meaningful roles in guiding families. In some cases, their early and experience-based explanations about the SPING procedure helped families navigate the decision process more confidently and coherently across care settings.

*"Yeah, you never really know if you’re doing the right thing, do you? [cries] So you do it with the best intentions, and you hope that it.. yeah, you hope it’s the right choice. And I think, looking back, it was probably an okay choice."*

## Emotional toll and coping with witnessing decline

### Emotional impact

Informal caregivers thoroughly expressed the emotional impact of illness and impending death. One patient explicitly expressed to his informal caregiver his desire to die. Relatives faced their own emotional struggles and were also subjected to profound grief, witnessing the patient suffer. Several caregivers described moments of deep emotional pain, sometimes breaking down in private or questioning whether they had made the right decisions.

*"For us, that was hard too. It almost felt like we were doing something worse to him, you know ..by not going ahead with the surgery. But we truly believed that surgery wasn’t an option. He was so unhappy in the hospital, he was.. yeah, it just wasn’t him.. really unsettled, confused. He had always been such a calm man. It was actually really hard to witness."*

### Coping with patients’ functional decline

Informal caregivers described the progression of physical decline of the patient before and after treatment as emotionally difficult, especially as their loved ones became more dependent on help. Loss of mobility and independence were common themes. Some caregivers had seen the decline coming over time, while for others it felt sudden, often triggered by the hip fracture. That moment was seen by many as a turning point, with the hip fracture symbolizing a loss of autonomy and overall quality of life. Particularly in cases of cognitive impairment, a lack of insight complicated both the caregiving experience and treatment decision-making, as patients were sometimes unable to grasp the full implications of their condition despite intermittent lucidity.

Over time, caregivers developed various coping strategies. Some adopted practical coping mechanisms such as managing daily tasks and maintaining self-reliance. Others processed their emotions and managed their stress, reflecting emotional coping. Some derived fulfillment and gratitude from their caregiver’s role, reflecting meaning-focused coping. The emotional burden of caregiving was consistently reported by all participants, with coping identified as an important and central aspect of the experience.

*“No, I didn’t get any professional help but that’s probably just my generation. You just sort of get through it on your own. At some point, you realize it’s up to you to pick up the pieces and keep going.”*

*“Looking back, it was the right choice. At the time, none of us were ready to say goodbye. I think that had a lot to do with it.”*

### Grieving and emotional aftermath

The final stage of life was experienced as a complex interplay of grief, acceptance, and in some cases, a sense of relief. The emotional burden of impending death was substantial, with frequently reported feelings including sadness and guilt. At the same time, a sense of acceptance and emotional calm gradually emerged for some, as death became imminent. The grieving process was described as challenging, often requiring a period of emotional adjustment and gradual acceptance.

Quality of palliative care during the final phase significantly shaped the overall experiences; some reported peaceful, symptom-controlled dying processes, while others found witnessing ongoing physical and emotional deterioration distressing. Throughout dignity, comfort, and support remained central to perceptions of care.

*“It’s part of life. Losing someone is never easy. Yes, one day they will lose me too… You have to come to terms with that.”*

*"My mum’s still alive, and honestly, she’s doing pretty well. But she keeps saying, 'I miss him so much.' I don’t think that feeling will ever go away."*

## Shifting roles and long-term caregiving burden

### Family dynamics

Several informal caregivers described the decision making as a process involving only the closest family members. These were usually those who had been designated as the primary decision-makers. They noted that discussions were often limited to a few siblings who had the legal authority to make decisions, and that consensus was reached relatively quickly among them. However, there were sometimes differences in perspective, particularly between those with more in-depth knowledge of the medical situation, which led to emotional tension. Despite these challenges, the shared experience of caring and the mutual concern for the patient's wellbeing fostered a strong sense of responsibility within the family.

*But it really comes down to the people closest to the situation at that moment. They’re the ones who need to understand what they’re deciding on. Because my mother couldn’t do it, and my father… well, he couldn’t make that decision either. He just said, ‘I’ll leave it to you girls.’"* [laughs]

### Shifting roles of family members

Informal caregivers described that the hip fracture significantly changed their family roles. Prior to the incident, caring responsibilities were sometimes already shared between siblings, often blurring the line between being a child and being an informal caregiver. The hip fracture further intensified this shift. One participant’s role as a caregiver inadvertently overwhelmed his role as a family member. He expressed mixed feelings about this dual responsibility. He reported that he had accepted the caring responsibilities, but the additional burden felt particularly challenging. After treatment and discharge to a nursing home, family members were able to resume a more traditional family role. Direct care responsibilities were reassigned. Overall, the experience highlighted the ongoing struggle to balance personal relationships with the demands of caring. It also emphasized the emotional impact of having to make difficult decisions on behalf of a loved one.

### Burden of care

Several caregivers described the burden of care before and after treatment as intense and multifaceted. The daily routine of informal care was often described as being on 'autopilot', with frequent visits and repeated moments of care being the norm. Although caregivers cared for their parents with love and dedication, they also experienced moments of exhaustion and need for escape, and the enjoyment of life for both care recipients and caregivers was affected. In case of a transition to professional care which provided some relief, but the long-term effects of caregiving were still felt.

Partners described having to make extensive practical adjustments in living arrangements and care routines, often without clear guidance or standardized protocols. This was frequently described as “reinventing the wheel.” This added to the caregivers’ burden and uncertainty. Caregivers reported that these adjustments required substantial energy and were maintained over several weeks. Caregivers navigated a complex balance between maintaining autonomy and increasing reliance on care. The choice for NOM often reflected a desire to avoid burdensome interventions, yet resulting in intensified dependence. Some informal caregivers described SPING not as part of NOM but as a second-choice intervention reserved for patients deemed too frail for surgery. They also mentioned to embrace SPING block to spare their loved one the risks and recovery demands of an operation.

*"Yeah, but it does take a lot of energy… yeah. And really, that’s how it had already been during those two months—well, just under two months—that my dad was still around… So yeah, his joy in life didn’t really improve, no."*

## Discussion

This qualitative study offers novel insights into how informal caregivers experience the decision-making process and care trajectory surrounding SPING block treatment in frail, older patients with hip fractures. Four core domains of caregiver experience emerged from thematic analysis: (1) Pain relief as a core treatment priority; (2) Complex decision-making and the caregiver’s moral burden; (3) Emotional toll and coping with witnessing decline; and (4) Shifting roles and long-term caregiving burden. Together, these findings provide a more nuanced understanding of the family experience in non-operative and palliative hip fracture care and suggest specific areas for clinical and systemic improvement.

Pain unequivocally emerged as the most pressing concern for informal caregivers, who reported that adequate pain relief was their highest priority. For them, uncontrolled pain was the biggest factor in their loved ones’ quality of life. This reflects earlier research identifying pain as a major determinant of comfort in patients with hip fractures receiving palliative care [24]. Healthcare professionals must anticipate this concern by offering timely, person-centered education and appropriate pain management strategies. The SPING block addressed this need effectively, providing substantial and sustained pain relief while reducing opioid use [14]. In addition to its analgesic value, early and effective pain control facilitated early discharge. This is crucial, given that prolonged hospitalization added both physical and emotional stress, especially for cognitively impaired patients. Family typically preferred their loved ones to return to their familiar environment as soon as possible. The median time from SPING block administration to hospital discharge is only one day [14]. This aligns with trends in geriatric palliative care, where early discharge and home-based care are often preferred to optimize remaining quality of life [25–27]. Clinically, this highlights the need for flexible protocols that support expedited discharge when appropriate. The rapid and sustained pain relief also promoted improving mobility from a supine to an upright position, contributing to perceived improvements in patient comfort. Earlier research shows that within 24 h of receiving the SPING block, 84% of patients can sit upright, and 44% can transfer from bed to chair, making early discharge a realistic and safe option [14].

Yet, the decision to proceed with SPING block was rarely straightforward. Informal caregivers emphasized the need for honest, empathetic communication regarding treatment options, prognosis and aftercare. They wanted clear explanations of how SPING block works, its risks and benefits compared to surgery or traditional pain medications, and realistic expectations about the recovery trajectory. This finding aligns with prior research indicating that both patients and families often have limited understanding of hip fracture prognosis and desire more timely information [28]. SDM emerged as crucial, caregivers valued being actively involved in discussions, having the patient’s values and goals centrally considered, and participating in advance care planning early in the process.

These findings underscore that SDM for SPING block frequently occurs under acute time pressure and emotional strain, with cognitive impairment frequently shifting the decision-making burden onto informal caregivers. Our results underscore the importance of initiating advance care planning discussions with frail older adults well before a health crisis, so that treatment preferences are documented and aligned with patient values. This could reduce the decisional and moral burden placed on families during emergencies.

Interestingly, informal caregivers did not express concerns regarding motor paresis following SPING block. This finding is clinically important, as it suggests that permanent motor impairment may be consciously accepted as a seen trade-off when balanced against the benefits of effective pain relief and comfort. In this study, motor paresis occurred in all patients, as this effect is an inherent and expected side effect of the SPING block. Importantly, this consequence was explicitly discussed with patients and caregivers in the during SDM, including clear communication about the permanent loss of motor function in the treated limb.

These findings contrast with the assumption commonly held by clinicians, often grounded in the principle of nonmaleficence, that motor paresis represents a major adverse outcome that should be avoided. From the caregiver perspective, however, the prioritization of comfort, relief of suffering, and the ability to rest or be positioned comfortably appeared to outweigh concerns about motor function. This highlights a potential mismatch between clinician- and caregiver-defined treatment priorities and underscores the importance of SDM that explicitly explores which outcomes matter most to patients and their families in palliative hip fracture care.

Palliative care guidelines similarly emphasize that effective SDM, including clear communication of prognosis and alternatives, is a cornerstone of care for frail, older patients [9]. A person-centered, multidisciplinary approach (involving patients, informal caregivers, trauma surgeons, geriatricians, anesthesiologists, and palliative care specialists) was viewed as necessary to tailor decisions to each patient’s situation and to ensure consistent messaging. When care teams were well-coordinated, caregivers felt more confident; conversely, poor coordination sometimes led to confusion or conflicting advice.

Caring for a frail, older adult in the final stage of life imposed a substantial emotional burden on informal caregivers. They described intense sadness, anxiety, and guilt about their role and the patient’s suffering, often feeling helpless as they witnessed physical and cognitive deterioration. Many struggled with anticipatory grief, a finding consistent with studies reporting significant emotional strain that can persist even after the patient’s death [27–29].

Literature emphasizes that psychological, emotional, and spiritual care should be routinely integrated for both patients and families [30]. Integrated structured psychosocial support into the care pathway, including timely counselling and access to support services, may not only alleviate caregiver burden but also enhance SDM and patient-centred outcomes.

Our findings indicated that hip fractures significantly impact family roles and decision-making processes. Before the injury occurred, families would often informally share caregiving duties alongside their other family responsibilities. However, the fracture elevates these tasks to the level of formal responsibilities, generating rapid consensus among legally authorized decision-makers but also emotional tension when medical expertise varies. Crucially, clear and cohesive communication from healthcare professionals empowered families, whereas fragmented guidance exacerbated confusion and stress. Even with professional care, relatives remained emotionally burdened in the long term and advocated for structured support, ranging from empathetic counselling to standardized care protocols.

This qualitative study offers unique insights into family perspectives, complementing existing quantitative evidence and guiding more patient and family-centered care. However, several limitations warrant consideration. Our sample was limited to caregivers willing to be interviewed, introducing potential selection bias. We mostly spoke with relatives of deceased patients, which may not reflect the experiences of other caregivers of survivors. Recall bias may also be a concern, as interviews occurred an average of nearly two years after SPING treatment (range 11–40 months). This long interval may have led to selective memories and affected the accuracy or completeness of the caregivers’ recollections, particularly regarding details of the treatment period. However, it may also have provided caregivers with an opportunity to reflect more broadly on the long-term impact of the treatment.

Amphia hospital’s routine use of the SPING block may limit transferability to settings where this intervention is not standard. Furthermore, the Dutch healthcare system, with its relatively broader use of non-operative management for hip fractures compared to international practice, may influence the extent to which the results of this study can be generalized. Finally, SDM about SPING block treatment is closely connected to the broader choice between nonoperative and operative management. This decision is inherently complex and carries significant consequences. Moreover, how families perceived and experienced the SPING process could be strongly influenced by their awareness and preparedness regarding the pre-existing palliative trajectory and limited life expectancy prior to the fracture.

Taken together, our findings suggest several ways clinicians can better support caregivers in the context of SPING block and palliative hip fracture care. First, pain control should remain the top priority. When patients have severe fracture pain and a limited life expectancy: early consideration of SPING block or similar definitive pain strategies aligns treatment with families’ desire for rapid comfort and mobilization. Second, care pathways should be adjusted to minimize delays. Close collaboration between orthopedic surgeons, geriatricians, anesthesiologists, and palliative care specialists can streamline decision-making and discharge. Multidisciplinary co-management is known to improve outcomes and should include clear protocols for SPING block use when indicated [30]. Third, clinicians must communicate proactively. Before proceeding with SPING block, the team should explain all options and potential outcomes in an understandable way, allowing time for questions. Honest conversations about prognosis and what to expect from recovery or decline, can prepare families and avoid false hope. However, sometimes patients or their families do not want to hear the full range of realistic options. In these cases, it is important to refer to the SDM framework, ensuring that discussions remain patient-centered while still respecting preferences and values. Advance care planning discussions preferably initiated as part of routine hip fracture care, can also clarify treatment goals in case recovery is not achievable. Finally, non-medical support should be arranged: social workers, chaplains, or counselors can meet with families to address fears, anxiety, or spiritual needs.

Given the exploratory nature and limited prior work in this area, further research should prospectively evaluate patient-centered outcomes after SPING block (pain scores, opioid use, quality of life, and satisfaction) compared with standard care, as well as long-term analgesia duration and neurologic sequelae. Future research should also investigate caregiver experiences using real-time interviews to reduce recall bias. Healthcare professionals involved in hip fracture care, including surgeons, geriatricians, anesthesiologists, nurses and palliative care teams, should be engaged to identify feasible interventions that address the emotional and practical burdens of caregivers. Where possible, patient experiences, directly or via proxy, should be captured to ensure truly patient centered care. Finally, examining how SPING block and other palliative interventions integrate into comprehensive hip fracture care remains an urgent priority.

## Data Availability

All data generated or analysed during this study are available from the corresponding author upon reasonable request.
